# Supplementary material for: Differential processing of HIV envelope glycans on the virus and soluble recombinant trimer
Source: Nat Commun. 2018 Sep 12;9:3693. doi: 10.1038/s41467-018-06121-4 (PMC6135743; doi:10.1038/s41467-018-06121-4)
Supplement: Supplementary file 1 — Supplementary Information [file 41467_2018_6121_MOESM1_ESM.pdf]

Supplementary Information for

Differential processing of HIV Envelope glycans on the virus and soluble recombinant trimer

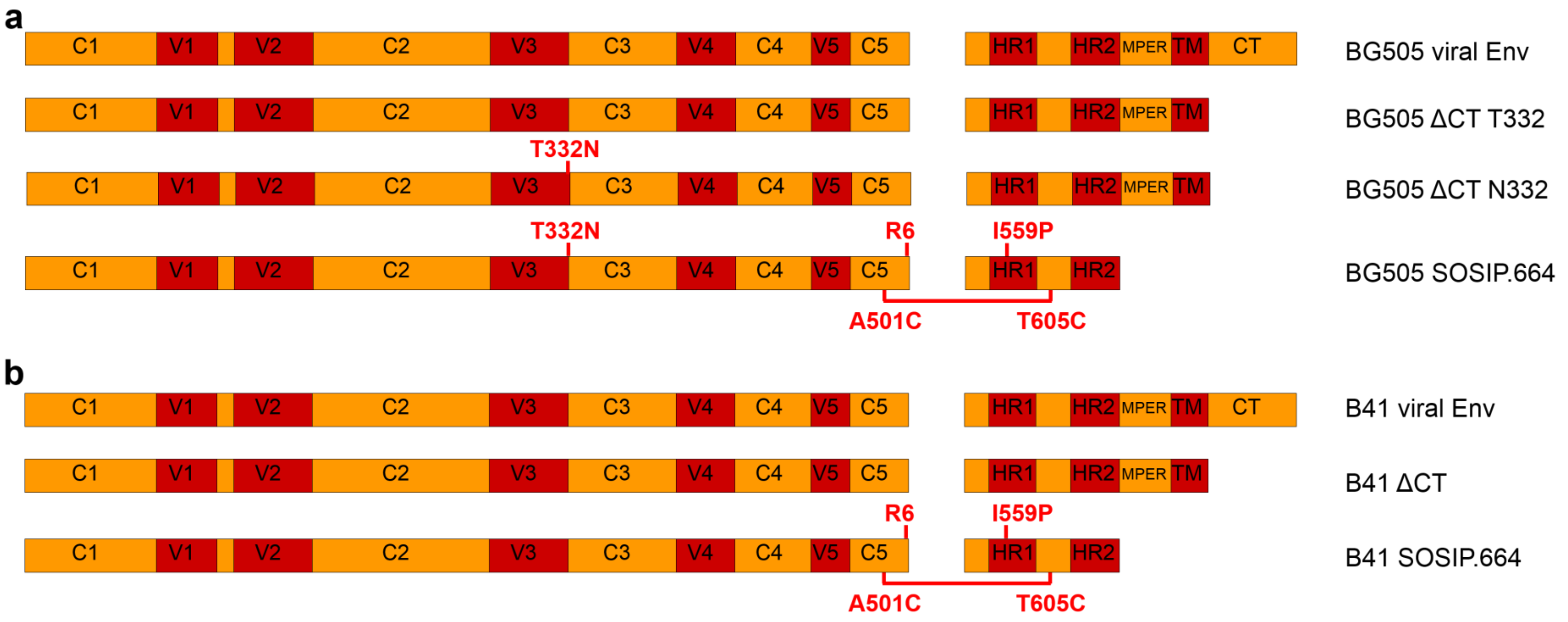

Supplementary Figure 1.
Design of the BG505 and B41 trimers.
Linear representation of (a) the BG505 viral Env, BG505 ΔCT T332, BG505 ΔCT N332, and BG505 SOSIP.664; (b) the B41 viral Env, B41 ΔCT, and B41 SOSIP.664. Modifications compared to the wild type sequences are indicated in red.

```
                                                     C1
JR-FL viral Env         WVTVYYGVPVWKEATTTLFCASDAKAYDTEVHNVWATHACVPTDPNPQE
JR-FL pseudovirus Env   WVTVYYGVPVWKEATTTLFCASDAKAYDTEVHNVWATHACVPTDPNPQE
JR-FL ΔCT               WVTVYYGVPVWKEATTTLFCASDAKAYDTEVHNVWATHACVPTDPNPQE
JR-FL SOSIP.664         WVTVYYGVPVWKEATTTLFCASDAKAYDTEVHNVWATHACVPTDPNPQE

                            88
JR-FL viral Env         VVLENVTEHFNMWKNNMVEQMQEDIISLWDQSLKPCVKLTPLCVTLNCK
JR-FL pseudovirus Env   VVLENVTEHFNMWKNNMVEQMQEDIISLWDQSLKPCVKLTPLCVTLNCK
JR-FL ΔCT               VVLENVTEHFNMWKNNMVEQMQEDIISLWDQSLKPCVKLTPLCVTLNCK
JR-FL SOSIP.664         VVLENVTEHFNMWKNNMVEQMQEDIISLWDQSLKPCVKLTPLCVTLNCK

                          135 138 141 V1         156 160         V2
JR-FL viral Env         DVNATNTTNDSEGTMERGEIKNCSFNITTSIRDEVQKEYALFYKLDVVP
JR-FL pseudovirus Env   DVNATNTTNDSEGTMERGEIKNCSFNITTSIRDEVQKEYALFYKLDVVP
JR-FL ΔCT               DVNATNTTNDSEGTMERGEIKNCSFNITTSIRDEVQKEYALFYKLDVVP
JR-FL SOSIP.664         DVNATNTTNDSEGTMERGEIKNCSFNITTSIRDKVQKEYALFYKLDVVP

                           186187                        C2
JR-FL viral Env         IDNNNTSYRLISCDTSVITQACPKISFEPIPIHYCAPAGFAILKCNDKT
JR-FL pseudovirus Env   IDNNNTSYRLISCDTSVITQACPKISFEPIPIHYCAPAGFAILKCNDKT
JR-FL ΔCT               IDNNNTSYRLISCDTSVITQACPKISFEPIPIHYCAPAGFAILKCNDKT
JR-FL SOSIP.664         IDNNNTSYRLISCDTSVITQACPKISFEPIPIHYCAPAGFAILKCNDKT

                                 241                 262           276
JR-FL viral Env         FNGKGPCKNVSTVQCTHGIRPVVSTQLLLNGSLAEEEVVIRSDNFTNNA
JR-FL pseudovirus Env   FNGKGPCKNVSTVQCTHGIRPVVSTQLLLNGSLAEEEVVIRSDNFTNNA
JR-FL ΔCT               FNGKGPCKNVSTVQCTHGIRPVVSTQLLLNGSLAEEEVVIRSDNFTNNA
JR-FL SOSIP.664         FNGKGPCKNVSTVQCTHGIRPVVSTQLLLNGSLAEEEVVIRSDNFTNNA

                                     295   301          V3
JR-FL viral Env         KTIIVQLKESVEINCTRPNNNTRKSIHIGPGRAFYTTGEIIGDIRQAHC
JR-FL pseudovirus Env   KTIIVQLKESVEINCTRPNNNTRKSIHIGPGRAFYTTGEIIGDIRQAHC
JR-FL ΔCT               KTIIVQLKESVEINCTRPNNNTRKSIHIGPGRAFYTTGEIIGDIRQAHC
JR-FL SOSIP.664         KTIIVQLKESVEINCTRPNNNTRKSIHIGPGRAFYTTGEIIGDIRQAHC

                        332    339              355 C3 362
JR-FL viral Env         NISRAKWNDTLKQIVIKLREQFENKTIVFNHSSGGDPEIVMHSFNCGGE
JR-FL pseudovirus Env   NISRAKWNDTLKQIVIKLREQFENKTIVFNHSSGGDPEIVMHSFNCGGE
JR-FL ΔCT               NISRAKWNDTLKQIVIKLREQFENKTIVFNHSSGGDPEIVMHSFNCGGE
JR-FL SOSIP.664         NISRAKWNDTLKQIVIKLREQFENKTIVFNHSSGGDPEIVMHSFNCGGE

                            386    392   397 V4 406                 C4
JR-FL viral Env         FFYCNSTQLFNSTWNNNTEGSNNTEGNTITLPCRIKQIINMWQEVGKAM
JR-FL pseudovirus Env   FFYCNSTQLFNSTWNNNTEGSNNTEGNTITLPCRIKQIINMWQEVGKAM
JR-FL ΔCT               FFYCNSTQLFNSTWNNNTEGSNNTEGNTITLPCRIKQIINMWQEVGKAM
JR-FL SOSIP.664         FFYCNSTQLFNSTWNNNTEGSNNTEGNTITLPCRIKQIINMWQEVGKAM

                                      448              463 V5         C4
JR-FL viral Env         YAPPIRGQIRCSSNITGLLLTRDGGINENGTEIFRPGGGDMRDNWRSEL
JR-FL pseudovirus Env   YAPPIRGQIRCSSNITGLLLTRDGGINENGTEIFRPGGGDMRDNWRSEL
JR-FL ΔCT               YAPPIRGQIRCSSNITGLLLTRDGGINENGTEIFRPGGGDMRDNWRSEL
JR-FL SOSIP.664         YAPPIRGQIRCSSNITGLLLTRDGGINENGTEIFRPGGGDMRDNWRSEL

                                          gp120 <-- | --> gp41
JR-FL viral Env         YKYKVVKIEPLGVAPTKAKRRVVQREKRAVGIGAVFLGFLGAAGSTMGA
JR-FL pseudovirus Env   YKYKVVKIEPLGVAPTKAKRRVVQREKRAVGIGAVFLGFLGAAGSTMGA
JR-FL ΔCT               YKYKVVKIEPLGVAPTKAKRRVVQREKRAVGIGAVFLGFLGAAGSTMGA
JR-FL SOSIP.664         YKYKVVKIEPLGVAPTKCKRRVRRRKKRAVGIGAVFLGFLGAAGSTMGA

                                                    HR1
JR-FL viral Env         ASMTLTVQARLLLSGIVQQQNNLLRAIEAQQRMLQLTVWGIKQLQARVL
JR-FL pseudovirus Env   ASMTLTVQARLLLSGIVQQQNNLLRAIEAQQRMLQLTVWGIKQLQARVL
JR-FL ΔCT               ASMTLTVQARLLLSGIVQQQNNLLRAIEAQQRMLQLTVWGIKQLQARVL
JR-FL SOSIP.664         ASMTLTVQARLLLSGIVQQQNNLLRAPEAQQRMLQLTVWGIKQLQARVL
```

```
                                                         611    616         625
JR-FL viral Env         AVERYLGDQQLLGIWGCSGKLICTTAVPWNASWSNKSLDRIWNNMTWME
JR-FL pseudovirus Env   AVERYLGDQQLLGIWGCSGKLICTTAVPWNASWSNKSLDRIWNNMTWME
JR-FL ΔCT               AVERYLGDQQLLGIWGCSGKLICTTAVPWNASWSNKSLDRIWNNMTWME
JR-FL SOSIP.664         AVERYLGDQQLLGIWGCSGKLICCTAVPWNASWSNKSLDRIWNNMTWME

                              637      HR2                          MPER
JR-FL viral Env         WEREIDNYTSEIYTLIEESQNQQEKNEQELLELDKWASLWNWFDITKWL
JR-FL pseudovirus Env   WEREIDNYTSEIYTLIEESQNQQEKNEQELLELDKWASLWNWFDITKWL
JR-FL ΔCT               WEREIDNYTSEIYTLIEESQNQQEKNEQELLELDKWASLWNWFDITKWL
JR-FL SOSIP.664         WEREIDNYTSEIYTLIEESQNQQEKNEQELLELGGGGHHHHHH------

                                         TM                          CT
JR-FL viral Env         WYIKIFIMIVGGLVGLRLVFTVLSIVNRVRQGYSPLSFQTLLPAPRGPD
JR-FL pseudovirus Env   WYIKIFIMIVGGLVGLRLVFTVLSIVNRVRQGYSPLSFQTLLPAPRGPD
JR-FL ΔCT               WYIKIFIMIVGGLVGLRLVFTVLSIVNRVRQGGGSGGGWSHPQFEK---
JR-FL SOSIP.664         -------------------------------------------------

JR-FL viral Env         RPEGIEEEGGERDRDRSGRLVNGFLALIWVDLRSLCLFSYHRLRDLLLT
JR-FL pseudovirus Env   RPEGIEEEGGERDRDRSGRLVNGFLALIWVDLRSLCLFSYHRLRDLLLT
JR-FL ΔCT               -------------------------------------------------
JR-FL SOSIP.664         -------------------------------------------------

JR-FL viral Env         VTRIVELLGRRGWEVLKYWWNLLQYWSQELKNSAVSLLNATAIAVAEGT
JR-FL pseudovirus Env   VTRIVELLGRRGWEVLKYWWNLLQYWSQELKNSAVSLLNATAIAVAEGT
JR-FL ΔCT               -------------------------------------------------
JR-FL SOSIP.664         -------------------------------------------------

JR-FL viral Env         DRIIEALQRTYRAILHIPTRIRQGLERALL
JR-FL pseudovirus Env   DRIIEALQRTYRAILHIPTRIRQGLERALL
JR-FL ΔCT               ------------------------------
JR-FL SOSIP.664         ------------------------------
```

Supplementary Figure 2.

Amino acid sequences of the JR-FL Env trimers analyzed in this study.

Sequence positions were aligned with the HIV reference strain HXB2 by using ClustalW multiple alignment. Potential N-glycosites are highlighted in red. Locations of different regions were indicated by using colored lines above sequences.

```
                                               C1
BG505 viral Env      AENLWVTVYYGVPVWKDAETTLFCASDAKAYETEKHNVWATHACVPTDP
BG505 ΔCT T332       AENLWVTVYYGVPVWKDAETTLFCASDAKAYETEKHNVWATHACVPTDP
BG505 ΔCT N332       AENLWVTVYYGVPVWKDAETTLFCASDAKAYETEKHNVWATHACVPTDP
BG505 SOSIP.664      AENLWVTVYYGVPVWKDAETTLFCASDAKAYETEKHNVWATHACVPTDP

                              88
BG505 viral Env      NPQEIHLENVTEEFNMWKNNMVEQMHTDIISLWDQSLKPCVKLTPLCVT
BG505 ΔCT T332       NPQEIHLENVTEEFNMWKNNMVEQMHTDIISLWDQSLKPCVKLTPLCVT
BG505 ΔCT N332       NPQEIHLENVTEEFNMWKNNMVEQMHTDIISLWDQSLKPCVKLTPLCVT
BG505 SOSIP.664      NPQEIHLENVTEEFNMWKNNMVEQMHTDIISLWDQSLKPCVKLTPLCVT

                        133 137 V1       156 160           V2
BG505 viral Env      LQCTNVTNNITDDMRGELKNCSFNMTTELRDKKQKVYSLFYRLDVVQIN
BG505 ΔCT T332       LQCTNVTNNITDDMRGELKNCSFNMTTELRDKKQKVYSLFYRLDVVQIN
BG505 ΔCT N332       LQCTNVTNNITDDMRGELKNCSFNMTTELRDKKQKVYSLFYRLDVVQIN
BG505 SOSIP.664      LQCTNVTNNITDDMRGELKNCSFNMTTELRDKKQKVYSLFYRLDVVQIN

                        185e 185h         197              C2
BG505 viral Env      ENQGNRSNNSNKEYRLINCNTSAITQACPKVSFEPIPIHYCAPAGFAIL
BG505 ΔCT T332       ENQGNRSNNSNKEYRLINCNTSAITQACPKVSFEPIPIHYCAPAGFAIL
BG505 ΔCT N332       ENQGNRSNNSNKEYRLINCNTSAITQACPKVSFEPIPIHYCAPAGFAIL
BG505 SOSIP.664      ENQGNRSNNSNKEYRLINCNTSAITQACPKVSFEPIPIHYCAPAGFAIL

                           234                             262
BG505 viral Env      KCKDKKFNGTGPCPSVSTVQCTHGIKPVVSTQLLLNGSLAEEEVMIRSE
BG505 ΔCT T332       KCKDKKFNGTGPCPSVSTVQCTHGIKPVVSTQLLLNGSLAEEEVMIRSE
BG505 ΔCT N332       KCKDKKFNGTGPCPSVSTVQCTHGIKPVVSTQLLLNGSLAEEEVMIRSE
BG505 SOSIP.664      KCKDKKFNGTGPCPSVSTVQCTHGIKPVVSTQLLLNGSLAEEEVMIRSE

                     276                295  301          V3
BG505 viral Env      NITNNAKNILVQFNTPVQINCTRPNNNTRKSIRIGPGQAFYATGDIIGD
BG505 ΔCT T332       NITNNAKNILVQFNTPVQINCTRPNNNTRKSIRIGPGQAFYATGDIIGD
BG505 ΔCT N332       NITNNAKNILVQFNTPVQINCTRPNNNTRKSIRIGPGQAFYATGDIIGD
BG505 SOSIP.664      NITNNAKNILVQFNTPVQINCTRPNNNTRKSIRIGPGQAFYATGDIIGD

                         332     339     C3   355      363
BG505 viral Env      IRQAHCTVSKATWNETLGKVVKQLRKHFGNNTIIRFANSSGGDLEVTTH
BG505 ΔCT T332       IRQAHCTVSKATWNETLGKVVKQLRKHFGNNTIIRFANSSGGDLEVTTH
BG505 ΔCT N332       IRQAHNTVSKATWNETLGKVVKQLRKHFGNNTIIRFANSSGGDLEVTTH
BG505 SOSIP.664      IRQAHNTVSKATWNETLGKVVKQLRKHFGNNTIIRFANSSGGDLEVTTH

                                386    392    398 V4 406  411     C4
BG505 viral Env      SFNCGGEFFYCNTSGLFNSTWISNTSVQGSNSTGSNDSITLPCRIKQII
BG505 ΔCT T332       SFNCGGEFFYCNTSGLFNSTWISNTSVQGSNSTGSNDSITLPCRIKQII
BG505 ΔCT N332       SFNCGGEFFYCNTSGLFNSTWISNTSVQGSNSTGSNDSITLPCRIKQII
BG505 SOSIP.664      SFNCGGEFFYCNTSGLFNSTWISNTSVQGSNSTGSNDSITLPCRIKQII

                                               448         462 V5
BG505 viral Env      NMWQRIGQAMYAPPIQGVIRCVSNITGLILTRDGGSTNSTTETFRPGGG
BG505 ΔCT T332       NMWQRIGQAMYAPPIQGVIRCVSNITGLILTRDGGSTNSTTETFRPGGG
BG505 ΔCT N332       NMWQRIGQAMYAPPIQGVIRCVSNITGLILTRDGGSTNSTTETFRPGGG
BG505 SOSIP.664      NMWQRIGQAMYAPPIQGVIRCVSNITGLILTRDGGSTNSTTETFRPGGG

                                C5                 gp120 <--|--> gp41
BG505 viral Env      DMRDNWRSELYKYKVVKIEPLGVAPTRAKRRVVGRE--KRAVGIGAVFL
BG505 ΔCT T332       DMRDNWRSELYKYKVVKIEPLGVAPTRAKRRVVGRE--KRAVGIGAVFL
BG505 ΔCT N332       DMRDNWRSELYKYKVVKIEPLGVAPTRAKRRVVGRE--KRAVGIGAVFL
BG505 SOSIP.664      DMRDNWRSELYKYKVVKIEPLGVAPTRCKRRVVGRRRRRRAVGIGAVFL

                                                         HR1
BG505 viral Env      GFLGAAGSTMGAASMTLTVQARNLLSGIVQQQSNLLRAIEAQQHLLKLT
BG505 ΔCT T332       GFLGAAGSTMGAASMTLTVQARNLLSGIVQQQSNLLRAIEAQQHLLKLT
BG505 ΔCT N332       GFLGAAGSTMGAASMTLTVQARNLLSGIVQQQSNLLRAIEAQQHLLKLT
BG505 SOSIP.664      GFLGAAGSTMGAASMTLTVQARNLLSGIVQQQSNLLRAPEAQQHLLKLT
```

```
                                                                                    611    618
BG505 viral Env      VWGIKQLQARVLAVERYLRDQQLLGIWGCSGKLICTTNVPWNSSWSNRN
BG505 ΔCT T332       VWGIKQLQARVLAVERYLRDQQLLGIWGCSGKLICTTNVPWNSSWSNRN
BG505 ΔCT N332       VWGIKQLQARVLAVERYLRDQQLLGIWGCSGKLICTTNVPWNSSWSNRN
BG505 SOSIP.664      VWGIKQLQARVLAVERYLRDQQLLGIWGCSGKLICCTNVPWNSSWSNRN

                            625         637    HR2
BG505 viral Env      LSEIWDNMTWLQWDKEISNYTQIIYGLLEESQNQQEKNEQDLLALDKWA
BG505 ΔCT T332       LSEIWDNMTWLQWDKEISNYTQIIYGLLEESQNQQEKNEQDLLALDKWA
BG505 ΔCT N332       LSEIWDNMTWLQWDKEISNYTQIIYGLLEESQNQQEKNEQDLLALDKWA
BG505 SOSIP.664      LSEIWDNMTWLQWDKEISNYTQIIYGLLEESQNQQEKNEQDLLALDGTK

                         MPER                    TM
BG505 viral Env      SLWNWFDISNWLWYIKIFIMIVGGLIGLRIVFAVLSVIHRVRQGYSPLS
BG505 ΔCT T332       SLWNWFDISNWLWYIKIFIMIVGGLIGLRIVFAVLSVIHRVRQGGGSGG
BG505 ΔCT N332       SLWNWFDISNWLWYIKIFIMIVGGLIGLRIVFAVLSVIHRVRQGGGSGG
BG505 SOSIP.664      HHHHHH-------------------------------------------

                                            CT
BG505 viral Env      FQTHTPNPRGLDRPERIEEEDGEQDRGRSTRLVSGFLALAWDDLRSLCL
BG505 ΔCT T332       GWSHPQFEK----------------------------------------
BG505 ΔCT N332       GWSHPQFEK----------------------------------------
BG505 SOSIP.664      -------------------------------------------------

BG505 viral Env      FCYHRLRDFILIAARIVELLGHSSLKGLRLGWEGLKYLWNLLAYWGREL
BG505 ΔCT T332       -------------------------------------------------
BG505 ΔCT N332       -------------------------------------------------
BG505 SOSIP.664      -------------------------------------------------

BG505 viral Env      KISAINLFDTIAIAVAEWTDRVIEIGQRLCRAFLHIPRRIRQGLERALL
BG505 ΔCT T332       -------------------------------------------------
BG505 ΔCT N332       -------------------------------------------------
BG505 SOSIP.664      -------------------------------------------------
```

Supplementary Figure 3.
Amino acid sequences of the BG505 Env trimers analyzed in this study.
Sequence positions were aligned with the HIV reference strain HXB2 by using ClustalW multiple alignment. Potential N-glycosites are highlighted in red. Locations of different regions were indicated by using colored lines above sequences.

```
                         C1
B41 viral Env    WVTVYYGVPVWKEATTTLFCASDAKAYDTEVHNVWATHACVPTDPNPQE
B41 ΔCT          WVTVYYGVPVWKEATTTLFCASDAKAYDTEVHNVWATHACVPTDPNPQE
B41 SOSIP.664    WVTVYYGVPVWKEATTTLFCASDAKAYDTEVHNVWATHACVPTDPNPQE

                     88
B41 viral Env    IVLGNVTENFNMWKNNMVEQMHEDIISLWDQSLKPCVKLTPLCVTLNCN
B41 ΔCT          IVLGNVTENFNMWKNNMVEQMHEDIISLWDQSLKPCVKLTPLCVTLNCN
B41 SOSIP.664    IVLGNVTENFNMWKNNMVEQMHEDIISLWDQSLKPCVKLTPLCVTLNCN

                     137140141143a V1         156 160      V2
B41 viral Env    NVNTNNTNNSTNATISDWEKMETGEMKNCSFNVTTSIRDKIKKEYALFY
B41 ΔCT          NVNTNNTNNSTNATISDWEKMETGEMKNCSFNVTTSIRDKIKKEYALFY
B41 SOSIP.664    NVNTNNTNNSTNATISDWEKMETGEMKNCSFNVTTSIRDKIKKEYALFY

                             186e 187     197          C2
B41 viral Env    KLDVVPLENKNNINNTNITNYRLINCNTSVITQACPKVSFEPIPIHYCA
B41 ΔCT          KLDVVPLENKNNINNTNITNYRLINCNTSVITQACPKVSFEPIPIHYCA
B41 SOSIP.664    KLDVVPLENKNNINNTNITNYRLINCNTSVITQACPKVSFEPIPIHYCA

                              234    241                   262
B41 viral Env    PAGFAILKCNSKTFNGSGPCTNVSTVQCTHGIRPVVSTQLLLNGSLAEE
B41 ΔCT          PAGFAILKCNSKTFNGSGPCTNVSTVQCTHGIRPVVSTQLLLNGSLAEE
B41 SOSIP.664    PAGFAILKCNSKTFNGSGPCTNVSTVQCTHGIRPVVSTQLLLNGSLAEE

                        276                  295    301   V3
B41 viral Env    EIVIRSENITDNAKTIIVQLNEAVEINCTRPNNNTRKSIHIGPGRAFYA
B41 ΔCT          EIVIRSENITDNAKTIIVQLNEAVEINCTRPNNNTRKSIHIGPGRAFYA
B41 SOSIP.664    EIVIRSENITDNAKTIIVQLNEAVEINCTRPNNNTRKSIHIGPGRAFYA

                             332     339       C3     355    362
B41 viral Env    TGDIIGNIRQAHCNISKARWNETLGQIVAKLEEQFPNKTIIFNHSSGGD
B41 ΔCT          TGDIIGNIRQAHCNISKARWNETLGQIVAKLEEQFPNKTIIFNHSSGGD
B41 SOSIP.664    TGDIIGNIRQAHCNISKARWNETLGQIVAKLEEQFPNKTIIFNHSSGGD

                                 386    392 396 V4           413
B41 viral Env    PEIVTHSFNCGGEFFYCNTTPLFNSTWNNTRTDDYPTGGEQNITLQCRI
B41 ΔCT          PEIVTHSFNCGGEFFYCNTTPLFNSTWNNTRTDDYPTGGEQNITLQCRI
B41 SOSIP.664    PEIVTHSFNCGGEFFYCNTTPLFNSTWNNTRTDDYPTGGEQNITLQCRI

                                    C4        448           463 V5
B41 viral Env    KQIINMWQGVGKAMYAPPIRGQIRCSSNITGLLLTRDGGRDQNGTETFR
B41 ΔCT          KQIINMWQGVGKAMYAPPIRGQIRCSSNITGLLLTRDGGRDQNGTETFR
B41 SOSIP.664    KQIINMWQGVGKAMYAPPIRGQIRCSSNITGLLLTRDGGRDQNGTETFR

                           C5                         gp120|gp41
B41 viral Env    PGGGNMRDNWRSELYKYKVVKIEPLGIAPTAAKRRVVQRE--KRAVGLG
B41 ΔCT          PGGGNMRDNWRSELYKYKVVKIEPLGIAPTAAKRRVVQRE--KRAVGLG
B41 SOSIP.664    PGGGNMRDNWRSELYKYKVVKIEPLGIAPTACKRRVVQRRRRRRAVGLG

                                                       HR1
B41 viral Env    AFILGFLGAAGSTMGAASMALTVQARLLLSGIVQQQNNLLRAIEAQQHM
B41 ΔCT          AFILGFLGAAGSTMGAASMALTVQARLLLSGIVQQQNNLLRAIEAQQHM
B41 SOSIP.664    AFILGFLGAAGSTMGAASMALTVQARLLLSGIVQQQNNLLRAPEAQQHM

                                                              611
B41 viral Env    LQLTVWGIKQLQARVLAVERYLRDQQLLGIWGCSGKIICTTNVPWNDSW
B41 ΔCT          LQLTVWGIKQLQARVLAVERYLRDQQLLGIWGCSGKIICTTNVPWNDSW
B41 SOSIP.664    LQLTVWGIKQLQARVLAVERYLRDQQLLGIWGCSGKIICCTNVPWNDSW

                 616     625          637 HR2
B41 viral Env    SNKTINEIWDNMTWMQWEKEIDNYTQHIYTLLEVSQIQQEKNEQELLEL
B41 ΔCT          SNKTINEIWDNMTWMQWEKEIDNYTQHIYTLLEVSQIQQEKNEQELLEL
B41 SOSIP.664    SNKTINEIWDNMTWMQWEKEIDNYTQHIYTLLEVSQIQQEKNEQELLEL

                       MPER                   TM
B41 viral Env    DKWDSLWNWFSISNWLWYIKIFIMIVGGLIGLRIVFTVLSIISRVRQGY
B41 ΔCT          DKWDSLWNWFSISNWLWYIKIFIMIVGGLIGLRIVFTVLSIISRVRQGG
B41 SOSIP.664    DGSGLNDIFEAQKIEWHE-------------------------------
```

```
                                             CT
B41 viral Env     SPLSFQTLLPVPRGPDRPEGIEEEGGERDRDRSGPPVNGFLAIFWVDLR
B41 ΔCT           GSGGGWSHPQFEK------------------------------------
B41 SOSIP.664     -------------------------------------------------

B41 viral Env     NLFLFLYHRLRDLLLIAARIVELLGRRGWGILKYWWNLLQYWSQELKNS
B41 ΔCT           -------------------------------------------------
B41 SOSIP.664     -------------------------------------------------

B41 viral Env     AVSLLNATAIAVAEGTDRVIEVVQRIVRGILHIPTRIRQGLERALL
B41 ΔCT           ----------------------------------------------
B41 SOSIP.664     ----------------------------------------------
```

Supplementary Figure 4.

Amino acid sequences of the B41 Env trimers analyzed in this study.

Sequence positions were aligned with the HIV reference strain HXB2 by using ClustalW multiple alignment. Potential N-glycosites are highlighted in red. Locations of different regions were indicated by using colored lines above sequences.

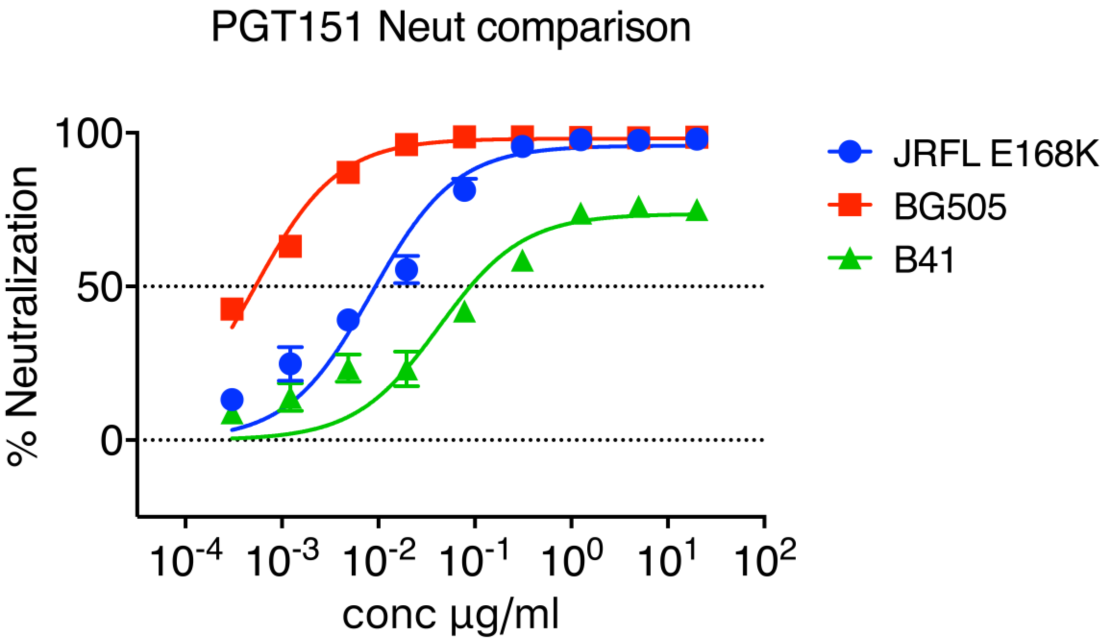

Supplementary Figure 5

Comparison of neutralization of PGT151 to the three viruses. Neutralization of the three viruses analyzed in this study (JR-FL E168K, BG505, and B41) by PGT151.

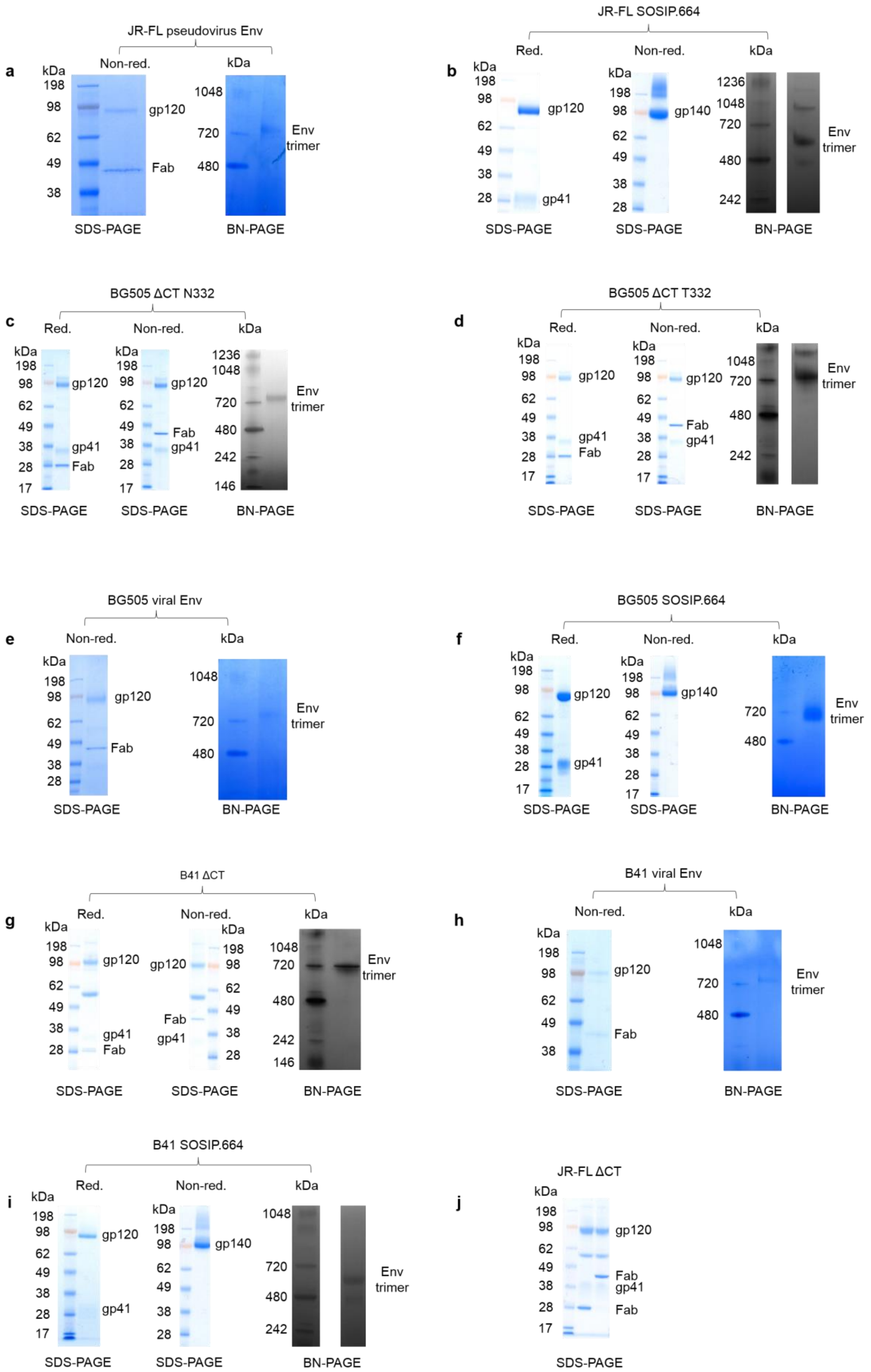

a
JR-FL pseudovirus Env
Non-red.
kDa
198
98
62
49
38
gp120
Fab
SDS-PAGE
kDa
1048
720
480
Env
trimer
BN-PAGE
b
JR-FL SOSIP.664
Red.
kDa
198
98
62
49
38
28
gp120
gp41
SDS-PAGE
Non-red.
kDa
198
98
62
49
38
28
gp140
SDS-PAGE
kDa
1236
1048
720
480
242
Env
trimer
BN-PAGE
c
BG505 ΔCT N332
Red.
kDa
198
98
62
49
38
28
17
gp120
gp41
Fab
SDS-PAGE
Non-red.
kDa
198
98
62
49
38
28
17
gp120
Fab
gp41
SDS-PAGE
kDa
1236
1048
720
480
242
146
Env
trimer
BN-PAGE
d
BG505 ΔCT T332
Red.
kDa
198
98
62
49
38
28
17
gp120
gp41
Fab
SDS-PAGE
Non-red.
kDa
198
98
62
49
38
28
17
gp120
Fab
gp41
SDS-PAGE
kDa
1048
720
480
242
Env
trimer
BN-PAGE
e
BG505 viral Env
Non-red.
kDa
198
98
62
49
38
28
gp120
Fab
SDS-PAGE
kDa
1048
720
480
Env
trimer
BN-PAGE
f
BG505 SOSIP.664
Red.
kDa
198
98
62
49
38
28
17
gp120
gp41
SDS-PAGE
Non-red.
kDa
198
98
62
49
38
28
17
gp140
SDS-PAGE
kDa
720
480
Env
trimer
BN-PAGE
g
B41 ΔCT
Red.
kDa
198
98
62
49
38
28
gp120
gp41
Fab
SDS-PAGE
Non-red.
kDa
198
98
62
49
38
28
gp120
Fab
gp41
SDS-PAGE
kDa
1048
720
480
242
146
Env
trimer
BN-PAGE
h
B41 viral Env
Non-red.
kDa
198
98
62
49
38
gp120
Fab
SDS-PAGE
kDa
1048
720
480
Env
trimer
BN-PAGE
i
B41 SOSIP.664
Red.
kDa
198
98
62
49
38
28
17
gp120
gp41
SDS-PAGE
Non-red.
kDa
198
98
62
49
38
28
gp140
SDS-PAGE
kDa
1048
720
480
242
Env
trimer
BN-PAGE
j
JR-FL ΔCT
kDa
198
98
62
49
38
28
17
gp120
Fab
gp41
Fab
SDS-PAGE

Supplementary Figure 6
Characterization of HIV-1 Env trimers analyzed in this study.
(a) SDS-PAGE (nonreducing) and BN-PAGE analysis of JR-FL pseudovirus Env; (b) SDS-PAGE (reducing and nonreducing) and BN-PAGE analysis of JR-FL SOSIP.664; (c) SDS-PAGE (reducing and nonreducing) and BN-PAGE analysis of BG505 ΔCT N332; (d) SDS-PAGE (reducing and nonreducing) and BN-PAGE analysis of BG505 ΔCT T332; (e) SDS-PAGE (nonreducing) and BN-PAGE analysis of BG505 viral Env; (f) SDS-PAGE (reducing and nonreducing) and BN-PAGE analysis of BG505 SOSIP.664; (g) SDS-PAGE (reducing and nonreducing) and BN-PAGE analysis of B41 ΔCT; (h) SDS-PAGE (nonreducing) and BN-PAGE analysis of B41 viral Env; (i) SDS-PAGE (reducing and nonreducing) and BN-PAGE analysis of B41 SOSIP.664. (j) Uncropped SDS-PAGE (reducing and nonreducing) of JR-FL ΔCT. The gp41 bands of JR-FL pseudovirus Env, BG505 viral Env, and B41 viral Env were not detected during SDS-PAGE analysis due to limited material.

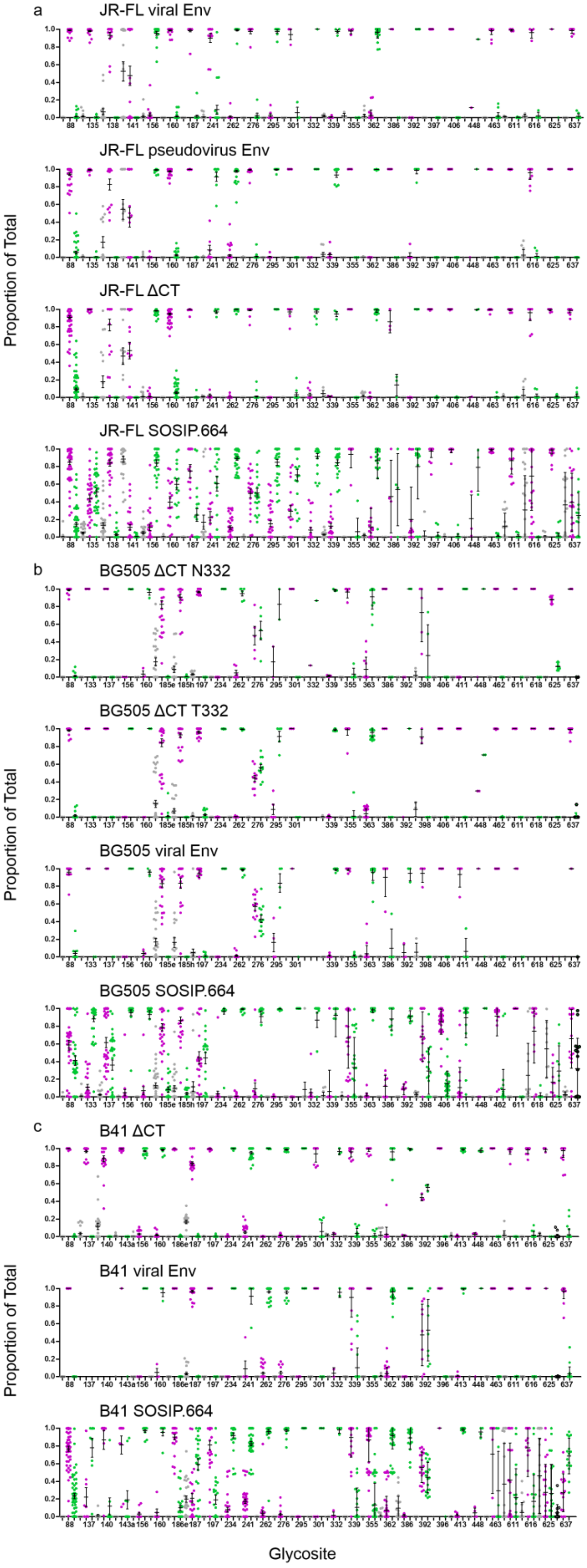
a
JR-FL viral Env
JR-FL pseudovirus Env
JR-FL ΔCT
JR-FL SOSIP.664
b
BG505 ΔCT N332
BG505 ΔCT T332
BG505 viral Env
BG505 SOSIP.664
c
B41 ΔCT
B41 viral Env
B41 SOSIP.664
Proportion of Total
Glycosite

Supplementary Figure 7.

Comparison of site-specific N-glycosylation of HIV-1 Env trimers, related to Figure 2.

Scatter plot of the site-specific glycosylation of (a) Env trimers from the JR-FL strain; (b) Env trimers from the BG505 strain; (c) Env trimers from the B41 strain. The glycosylation profiles of JR-FL viral Env, BG505 ΔCT N332 Env, and B41 ΔCT Env were used as a reference for comparison with other Envs from the corresponding strain. Each recombinant Env was digested and analyzed in duplicate from two biological batches, while native trimers from infectious virus and pseudovirus were analyzed in duplicate from same biological preparation (n=6). Mean ± s.e.m. were plotted. Grey circle: no glycan, purple circle: complex type, and green circle: high mannose.

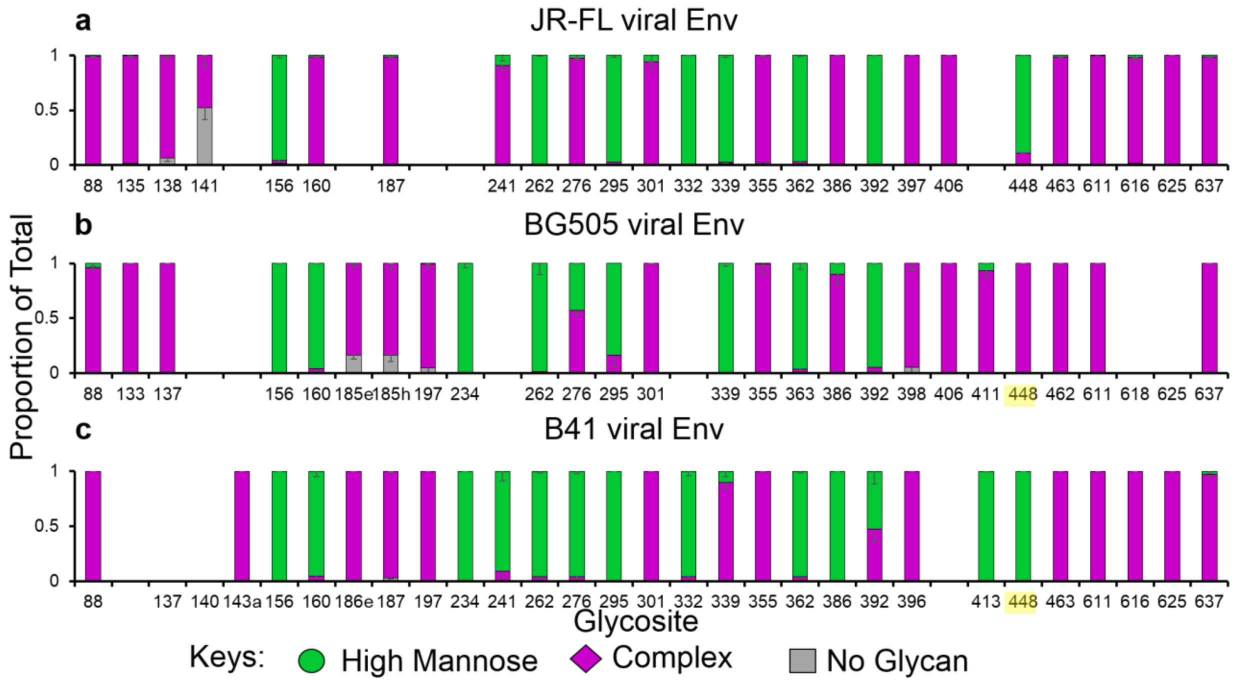

Supplementary Figure 8.

Comparison of site-specific N-glycan processing of Env trimers derived from different HIV-1 infectious viruses.

Site-specific N-glycan processing of well-ordered, cleaved trimeric Env from different HIV infectious viruses, including (a) JR-FL, (b) BG505, and (c) B41. BG505 virus has the original BG505 gp160 sequence and does not have glycosite at the position 332. N-glycosites from multiple HIV Env trimers were aligned according to their relative position to HXB2 and their predicted functional importance. The proportions of high-mannose and complex-type glycans at the glycosites highlighted in yellow were assigned based on the proportion of spectra hits, since respective peak areas did not reach the threshold. The N137 and N140 glycans in B41 virus Env and the N618 and N625 glycans in BG505 viral Env were not detected. The N448 glycan in BG505 viral Env was detected with only one spectrum hit. Thus, those glycosites were not included in the comparison. Native trimers from infectious virus were digested and analyzed in duplicate from same biological preparation (n=6). Differences were assessed for the proportions of no glycan, high mannose and complex type at each glycosite. Differences of >10% were determined to be significant if the *P* value was <0.05 using a Mann-Whitney test. Mean ± s.e.m. were plotted.

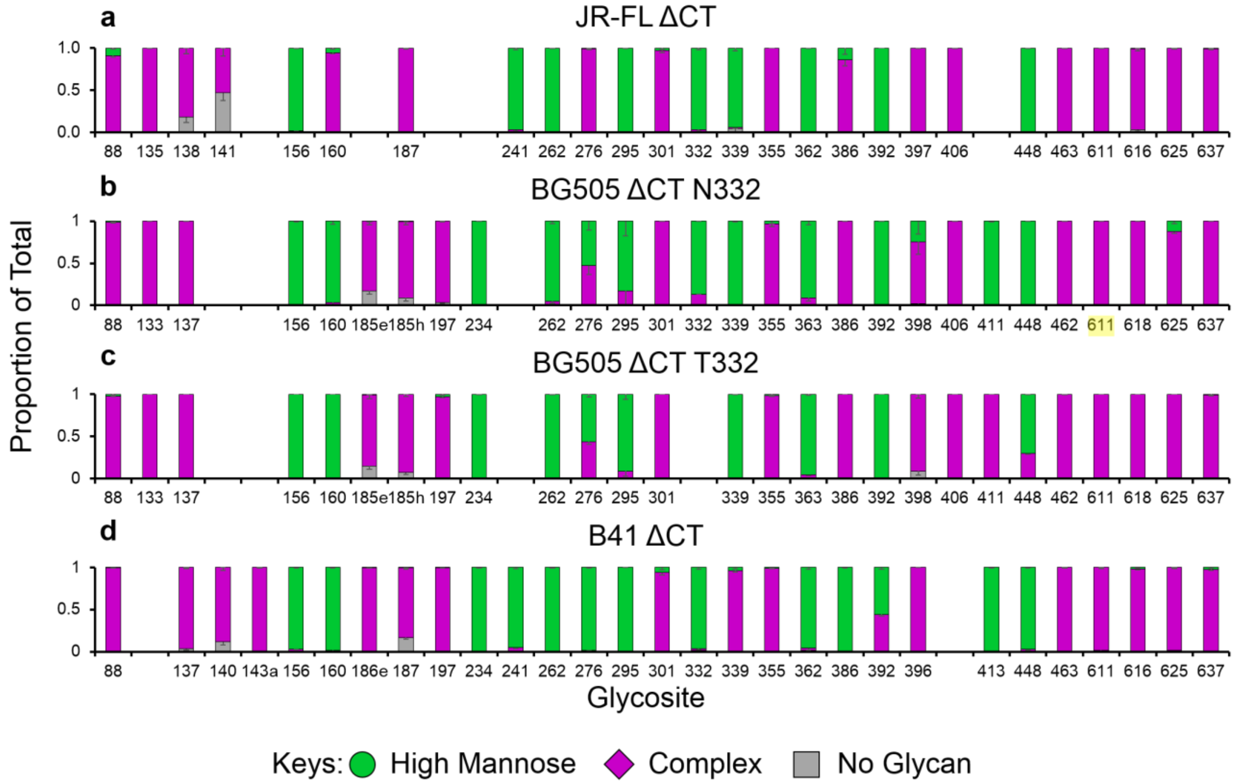

Supplementary Figure 9.

Comparison of site-specific N-glycan processing of recombinant membrane-bound Env trimers from different HIV-1 isolates.

Site-specific N-glycan processing of (a) JR-FL ΔCT, (b) BG505 ΔCT N332, (c) BG505 ΔCT T332, (d) B41 ΔCT. N-glycosites from multiple HIV Env trimers were aligned according to their relative position to HXB2 and their predicted functional importance. The proportions of high-mannose and complex-type glycans at the glycosites highlighted in yellow were assigned based on the proportion of spectra hits, since respective peak area did not reach the threshold. Each recombinant Env was digested and analyzed in duplicate from two biological batches (n=6). Differences were assessed for the proportions of no glycan, high mannose and complex type at each glycosite. Differences of >10% were determined to be significant if the *P* value was <0.05 using a Mann-Whitney test. Mean ± s.e.m. were plotted.

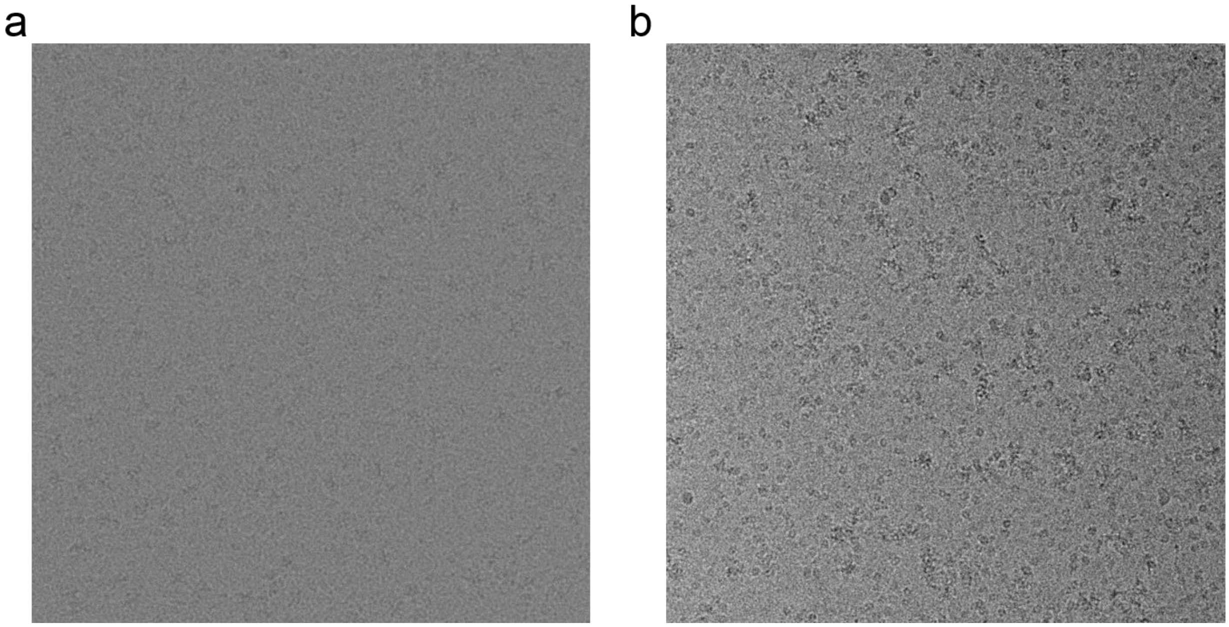

Supplementary Figure 10.
Representative EM micrographs.
Representative EM micrographs of (a) BG505 ΔCT T332–PGT151 frozen on a continuous carbon substrate and (b) B41 ΔCT-PGT151 frozen on a holey carbon grid.

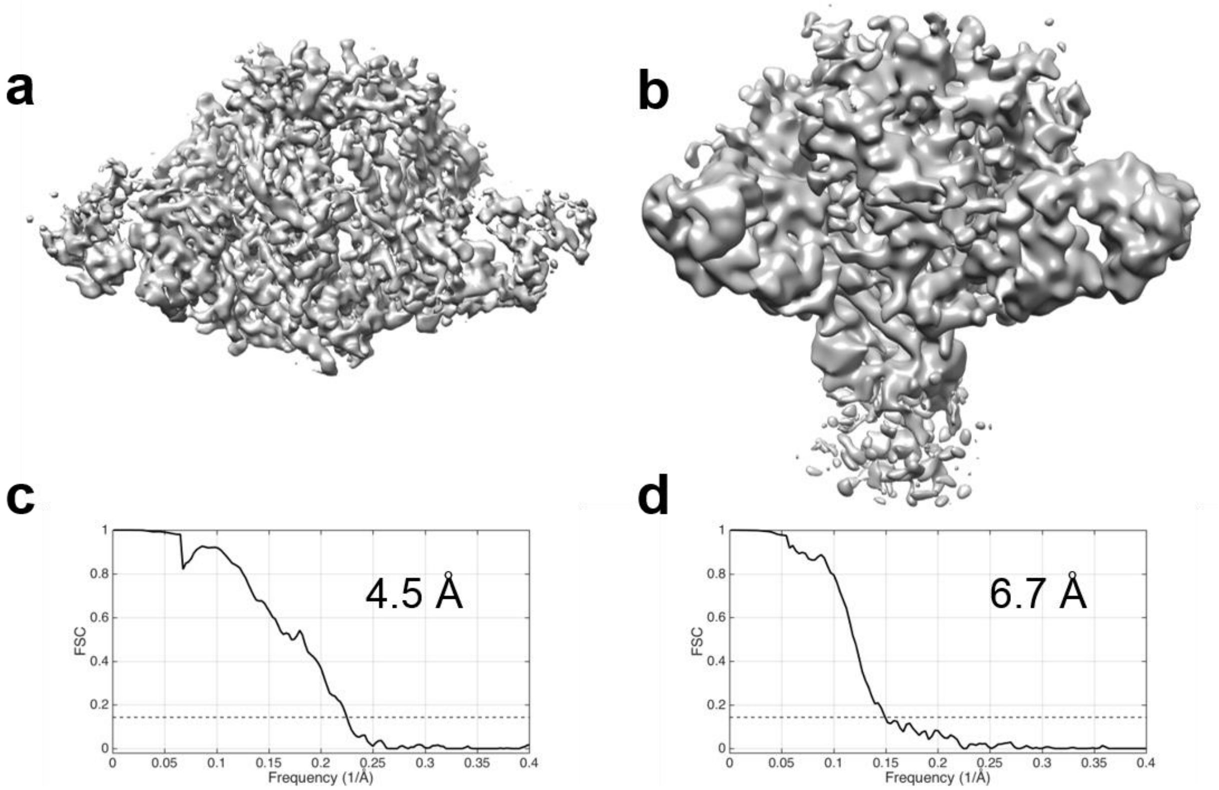

Supplementary Figure 11.

Fourier shell correlation curves for Cryo-EM maps of BG505 ΔCT T332-PGT151 and B41 ΔCT-PGT151.

Cryo-EM density maps (a, b) and corresponding Fourier shell correlation (FSC) plots (c, d) showing resolution where the gold-standard FSC drops below 0.143.

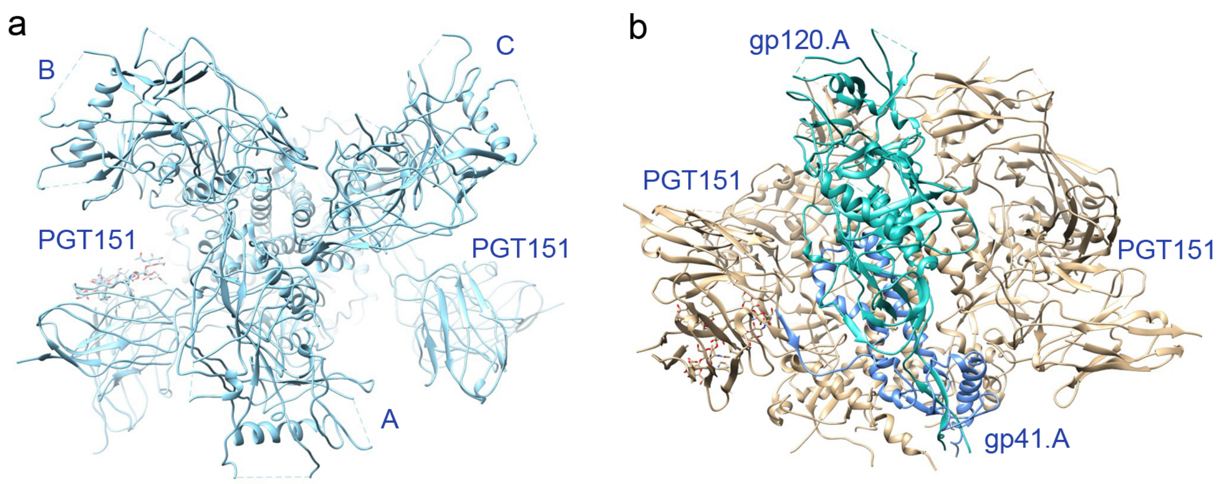

c

BG505 ΔCT: new model	BG505 SOSIP.664: 5ACO	Cα RMSD
ΔCT.gp120.A	SOSIP.gp120	2.126
ΔCT.gp120.B	SOSIP.gp120	2.117
ΔCT.gp120.C	SOSIP.gp120	2.197
ΔCT.gp41.A	SOSIP.gp41	1.812
ΔCT.gp41.B	SOSIP.gp41	2.350
ΔCT.gp41.C	SOSIP.gp41	2.411

d

JR-FL ΔCT: 5FUU	JR-FL SOSIP.664: 5FYK	Cα RMSD
ΔCT.gp120.A	SOSIP.gp120	1.300
ΔCT.gp120.B	SOSIP.gp120	1.395
ΔCT.gp120.C	SOSIP.gp120	1.486
ΔCT.gp41.A	SOSIP.gp41	3.888
ΔCT.gp41.B	SOSIP.gp41	3.065
ΔCT.gp41.C	SOSIP.gp41	3.797

e

BG505 ΔCT: new model	JR-FL ΔCT: 5FUU	Cα RMSD
ΔCT.gp120.A	ΔCT.gp120.A	1.595
ΔCT.gp120.B	ΔCT.gp120.B	1.702
ΔCT.gp120.C	ΔCT.gp120.C	1.845
ΔCT.gp41.A	ΔCT.gp41.A	1.500
ΔCT.gp41.B	ΔCT.gp41.B	2.084
ΔCT.gp41.C	ΔCT.gp41.C	1.541

f

BG505 SOSIP.664: 5ACO	JR-FL SOSIP.664: 5FYK	Cα RMSD
SOSIP.gp120	SOSIP.gp120	1.439
SOSIP.gp41	SOSIP.gp41	1.918

Supplementary Figure 12.
Structural differences between recombinant membrane-bound and soluble SOSIP trimers.
The high-resolution structures of (a) JR-FL ΔCT (PDB id: 5FUU) and (b) JR-FL SOSIP.664 (PDB id: 5FYK). The three protomers of JR-FL ΔCT was labeled as protomer A, protomer B, and protomer C. Pair-wise Cα RMSDs for superimpositions between (c) BG505 ΔCT T332 and BG505 SOSIP.664, (d) JR-FL ΔCT and JR-FL SOSIP.664, (e) BG505 ΔCT T332 and JR-FL

ΔCT, and (F) BG505 SOSIP.664 and JR-FL SOSIP.664. All Cα RMSDs values were calculated one chain at a time with UCSF Chimera MatchMaker algorithm without “pruning”.

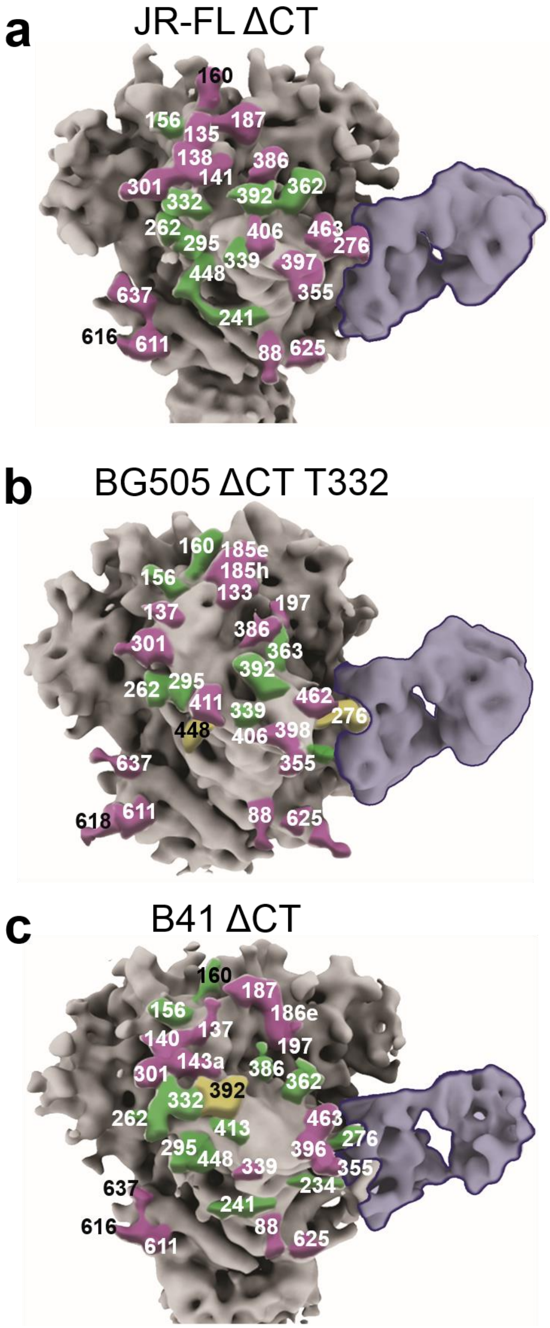

Supplementary Figure 13.
Mapping of site-specific glycan processing of recombinant membrane-bound trimers onto the surface of corresponding cryo-EM density maps.
Side view of high mannose patch of (a) JR-FL ΔCT, (b) BG505 ΔCT T332, (c) B41 ΔCT, related to Figure 4E. Note that only the glycans on one protomer were highlighted.

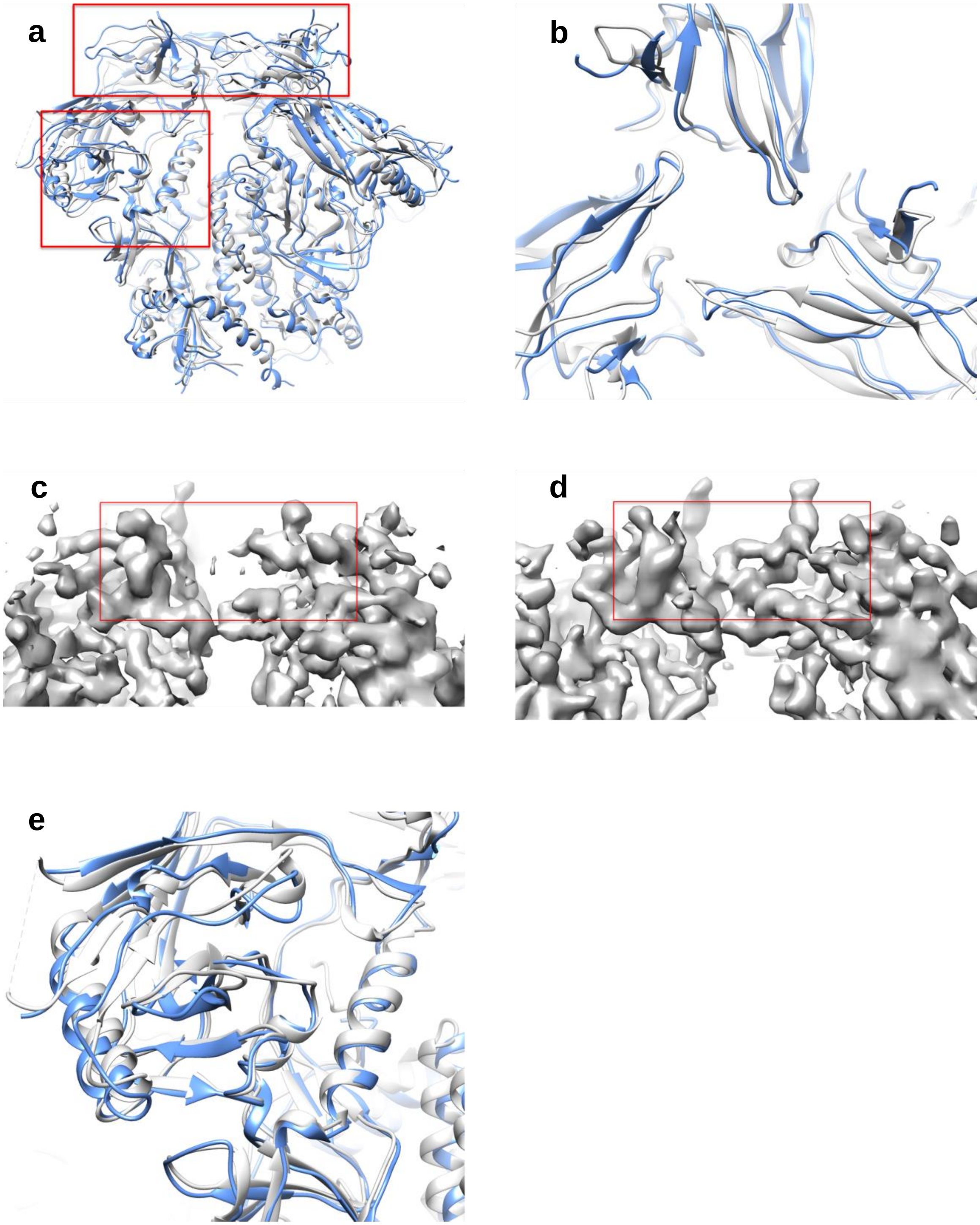

Supplementary Figure 14.
Comparison of cryo-EM reconstructions of BG505 ΔCT T332 and JR-FL ΔCT.
(a) Superimposition of the PGT151-bound JR-FL ΔCT (light gray) and BG505 ΔCT T332 (light blue). Boxed regions were shown in b and e. (b) Top view of Apex region boxed out in (a). (c) EM density of Apex region of JR-FL ΔCT (side view). (d) EM density of Apex region of BG505 ΔCT T332 (side view). (e) Side view of CD4 binding site boxed out in (a).

Supplementary Table 1.
The number of MS/MS spectra that could be detected from each glycosylation site of JR-FL viral Env, related to **Fig. 2a**.

Glycosite	N+0	N+3	N+203	Sum
88	37	871	93	1001
135	25	369	26	420
138	102	307	11	420
141	272	137	10	419
156	52	50	129	231
160	1	236	30	267
187	0	264	12	276
241	9	108	42	159
262	0	115	749	864
276	0	544	111	655
295	0	4	44	48
301	0	13	7	20
332	0	2	11	13
339	37	17	15	69
355	4	73	0	77
362	33	45	356	434
386	0	3	0	3
392	0	0	15	15
397	0	18	0	18
406	0	23	0	23
448	0	2	0	2
463	0	376	11	387
611	4	52	1	57
616	9	53	7	69
625	0	12	1	13
637	0	37	8	45

Supplementary Table 2.
Site-specific glycosylation of JR-FL viral Env, related to **Fig. 2a**.

Glycosite	N+0	N+3	N+203	Standard Error (N+0)	Standard Error (N+3)	Standard Error (N+203)
88	0.0002	0.9895	0.0103	0.0002	0.0043	0.0042
135	0.0144	0.9800	0.0056	0.0070	0.0094	0.0029
138	0.0668	0.9331	0.0001	0.0338	0.0339	0.0001
141	0.5241	0.4759	0.0000	0.1090	0.1090	0.0000
156	0.0143	0.0322	0.9535	0.0116	0.0206	0.0225
160	0.0000	0.9871	0.0129	0.0000	0.0063	0.0063
187	0.0000	0.9853	0.0147	0.0000	0.0147	0.0147
241	0.0085	0.9004	0.0911	0.0049	0.0513	0.0504
262	0.0000	0.0099	0.9901	0.0000	0.0091	0.0091
276	0.0000	0.9735	0.0265	0.0000	0.0254	0.0254
295	0.0000	0.0246	0.9754	0.0000	0.0181	0.0181
301	0.0000	0.9412	0.0588	0.0000	0.0587	0.0587
332	0.0000	0.0000	1.0000	0.0000	0.0000	0.0000
339	0.0177	0.0071	0.9752	0.0107	0.0071	0.0169
355	0.0204	0.9796	0.0000	0.0148	0.0148	0.0000
362	0.0104	0.0249	0.9647	0.0058	0.0117	0.0125
386	0.0000	1.0000	0.0000	0.0000	0.0000	0.0000
392	0.0000	0.0000	1.0000	0.0000	0.0000	0.0000
397	0.0000	1.0000	0.0000	0.0000	0.0000	0.0000
406	0.0000	1.0000	0.0000	0.0000	0.0000	0.0000
448	0.0000	0.1131	0.8869	0.0000	0.0000	0.0000
463	0.0000	0.9849	0.0151	0.0000	0.0096	0.0096
611	0.0064	0.9912	0.0023	0.0040	0.0050	0.0022
616	0.0150	0.9633	0.0216	0.0115	0.0321	0.0208
625	0.0000	1.0000	0.0000	0.0000	0.0000	0.0000
637	0.0000	0.9851	0.0149	0.0000	0.0102	0.0102

Supplementary Table 3.
Site-specific glycosylation of JR-FL pseudoviral Env, related to **Fig. 2a**.

Glycosite	N+0	N+3	N+203	Standard Error (N+0)	Standard Error (N+3)	Standard Error (N+203)
88	0.0000	0.9442	0.0558	0.0000	0.0165	0.0165
135	0.0042	0.9908	0.0050	0.0022	0.0050	0.0048
138	0.1737	0.8263	0.0000	0.0627	0.0627	0.0000
141	0.5432	0.4568	0.0000	0.1110	0.1110	0.0000
156	0.0048	0.0029	0.9924	0.0024	0.0023	0.0033
160	0.0000	0.9764	0.0236	0.0000	0.0077	0.0077
187	0.0000	0.9985	0.0015	0.0000	0.0006	0.0006
241	0.0021	0.0860	0.9119	0.0021	0.0523	0.0519
262	0.0000	0.0235	0.9765	0.0000	0.0097	0.0097
276	0.0004	0.9917	0.0079	0.0003	0.0054	0.0051
295	0.0000	0.0055	0.9945	0.0000	0.0054	0.0054
301	0.0000	1.0000	0.0000	0.0000	0.0000	0.0000
332	0.0000	0.0000	1.0000	0.0000	0.0000	0.0000
339	0.0346	0.0288	0.9365	0.0214	0.0191	0.0300
355	0.0000	1.0000	0.0000	0.0000	0.0000	0.0000
362	0.0012	0.0002	0.9986	0.0006	0.0001	0.0007
386	0.0000	1.0000	0.0000	0.0000	0.0000	0.0000
392	0.0000	0.0109	0.9891	0.0000	0.0109	0.0109
397	0.0000	1.0000	0.0000	0.0000	0.0000	0.0000
406	0.0000	1.0000	0.0000	0.0000	0.0000	0.0000
448	0.0000	0.0000	1.0000	0.0000	0.0000	0.0000
463	0.0000	0.9998	0.0002	0.0000	0.0002	0.0002
611	0.0004	0.9993	0.0003	0.0002	0.0005	0.0003
616	0.0272	0.9614	0.0115	0.0150	0.0192	0.0080
625	0.0000	1.0000	0.0000	0.0000	0.0000	0.0000
637	0.0000	1.0000	0.0000	0.0000	0.0000	0.0000

Supplementary Table 4.
Site-specific glycosylation of JR-FL ΔCT, related to **Fig. 2a**.

Glycosite	N+0	N+3	N+203	Standard Error (N+0)	Standard Error (N+3)	Standard Error (N+203)
88	0.0000	0.9087	0.0913	0.0000	0.0163	0.0163
135	0.0039	0.9958	0.0003	0.0022	0.0023	0.0001
138	0.1780	0.8220	0.0000	0.0647	0.0647	0.0000
141	0.4679	0.5321	0.0000	0.0933	0.0933	0.0000
156	0.0084	0.0091	0.9826	0.0040	0.0037	0.0053
160	0.0000	0.9456	0.0544	0.0000	0.0119	0.0119
187	0.0000	0.9969	0.0031	0.0000	0.0022	0.0022
241	0.0079	0.0217	0.9704	0.0049	0.0170	0.0165
262	0.0000	0.0022	0.9978	0.0000	0.0011	0.0011
276	0.0000	0.9898	0.0102	0.0000	0.0075	0.0075
295	0.0001	0.0001	0.9998	0.0001	0.0001	0.0002
301	0.0000	0.9701	0.0299	0.0000	0.0226	0.0226
332	0.0000	0.0300	0.9700	0.0000	0.0167	0.0167
339	0.0438	0.0102	0.9459	0.0353	0.0052	0.0301
355	0.0000	1.0000	0.0000	0.0000	0.0000	0.0000
362	0.0073	0.0016	0.9911	0.0027	0.0013	0.0036
386	0.0000	0.8590	0.1410	0.0000	0.0716	0.0716
392	0.0000	0.0000	1.0000	0.0000	0.0000	0.0000
397	0.0000	0.9994	0.0006	0.0000	0.0006	0.0006
406	0.0000	0.9997	0.0003	0.0000	0.0003	0.0003
448	0.0000	0.0036	0.9964	0.0000	0.0036	0.0036
463	0.0000	0.9985	0.0015	0.0000	0.0012	0.0012
611	0.0040	0.9956	0.0005	0.0027	0.0030	0.0003
616	0.0268	0.9630	0.0102	0.0150	0.0216	0.0070
625	0.0000	0.9960	0.0040	0.0000	0.0028	0.0028
637	0.0000	0.9888	0.0112	0.0000	0.0062	0.0062

Supplementary Table 5.
Site-specific glycosylation of JR-FL SOSIP.664, related to **Fig. 2a**.

Glycosite	N+0	N+3	N+203	Standard Error (N+0)	Standard Error (N+3)	Standard Error (N+203)
88	0.0049	0.8493	0.1459	0.0034	0.0268	0.0254
135	0.0484	0.4384	0.5133	0.0106	0.0411	0.0433
138	0.1338	0.8387	0.0275	0.0323	0.0363	0.0107
141	0.8824	0.1114	0.0062	0.0336	0.0327	0.0028
156	0.0477	0.1112	0.8411	0.0138	0.0357	0.0353
160	0.0040	0.3977	0.5983	0.0026	0.0621	0.0615
187	0.0004	0.7494	0.2502	0.0003	0.0732	0.0730
241	0.1695	0.2246	0.6059	0.0811	0.0654	0.0847
262	0.0000	0.1093	0.8907	0.0000	0.0210	0.0210
276	0.0025	0.4949	0.5026	0.0016	0.0502	0.0495
295	0.0003	0.1523	0.8474	0.0001	0.0335	0.0335
301	0.0003	0.2954	0.7043	0.0003	0.0659	0.0660
332	0.0000	0.0817	0.9183	0.0000	0.0298	0.0298
339	0.0294	0.1260	0.8446	0.0067	0.0290	0.0323
355	0.0000	0.9397	0.0603	0.0000	0.0587	0.0587
362	0.0155	0.1176	0.8669	0.0033	0.0463	0.0463
386	0.0000	0.4616	0.5384	0.0000	0.2054	0.2054
392	0.0000	0.1995	0.8005	0.0000	0.0872	0.0872
397	0.0122	0.9743	0.0134	0.0121	0.0144	0.0087
406	0.0014	0.9918	0.0068	0.0012	0.0065	0.0054
448	0.0000	0.2088	0.7912	0.0000	0.1556	0.1556
463	0.0000	0.9919	0.0080	0.0000	0.0022	0.0022
611	0.1261	0.8665	0.0074	0.0373	0.0394	0.0051
616	0.3085	0.6913	0.0002	0.0687	0.0687	0.0002
625	0.0029	0.9862	0.0109	0.0020	0.0066	0.0055
637	0.3642	0.3931	0.2427	0.0827	0.0736	0.0578

Supplementary Table 6.
Site-specific glycosylation of BG505 ΔCT N332, related to **Fig. 2b**.

Glycosite	N+0	N+3	N+203	Standard Error (N+0)	Standard Error (N+3)	Standard Error (N+203)
88	0.0000	0.9876	0.0124	0.0000	0.0072	0.0072
133	0.0000	1.0000	0.0000	0.0000	0.0000	0.0000
137	0.0000	1.0000	0.0000	0.0000	0.0000	0.0000
156	0.0000	0.0017	0.9983	0.0000	0.0008	0.0008
160	0.0000	0.0369	0.9631	0.0000	0.0332	0.0332
185e	0.1736	0.8251	0.0013	0.0424	0.0427	0.0006
185h	0.0890	0.9072	0.0037	0.0358	0.0366	0.0021
197	0.0328	0.9672	0.0000	0.0091	0.0091	0.0000
234	0.0000	0.0000	1.0000	0.0000	0.0000	0.0000
262	0.0000	0.0502	0.9498	0.0000	0.0277	0.0277
276	0.0000	0.4727	0.5273	0.0000	0.1057	0.1057
295	0.0000	0.1742	0.8258	0.0000	0.1721	0.1721
301	0.0000	1.0000	0.0000	0.0000	0.0000	0.0000
332	0.0000	0.1334	0.8666	0.0000	0.0000	0.0000
339	0.0004	0.0126	0.9870	0.0003	0.0066	0.0067
355	0.0000	0.9657	0.0343	0.0000	0.0301	0.0300
363	0.0017	0.0881	0.9101	0.0008	0.0404	0.0401
386	0.0000	1.0000	0.0000	0.0000	0.0000	0.0000
392	0.0000	0.0000	1.0000	0.0000	0.0000	0.0000
398	0.0204	0.7343	0.2452	0.0204	0.1483	0.1552
406	0.0000	1.0000	0.0000	0.0000	0.0000	0.0000
411	0.0000	0.0000	1.0000	0.0000	0.0000	0.0000
448	0.0000	0.0000	1.0000	0.0000	0.0000	0.0000
462	0.0000	1.0000	0.0000	0.0000	0.0000	0.0000
611	0.0000	1.0000	0.0000	0.0000	0.0000	0.0000
618	0.0000	1.0000	0.0000	0.0000	0.0000	0.0000
625	0.0000	0.8778	0.1222	0.0000	0.0272	0.0272
637	0.0000	1.0000	0.0000	0.0000	0.0000	0.0000

Supplementary Table 7.
Site-specific glycosylation of BG505 ΔCT T332, related to **Fig. 2b**.

Glycosite	N+0	N+3	N+203	Standard Error (N+0)	Standard Error (N+3)	Standard Error (N+203)
88	0.0000	0.9816	0.0184	0.0000	0.0094	0.0094
133	0.0000	1.0000	0.0000	0.0000	0.0000	0.0000
137	0.0000	1.0000	0.0000	0.0000	0.0000	0.0000
156	0.0000	0.0000	1.0000	0.0000	0.0000	0.0000
160	0.0000	0.0000	1.0000	0.0000	0.0000	0.0000
185e	0.1499	0.8405	0.0096	0.0413	0.0448	0.0076
185h	0.0700	0.9300	0.0000	0.0237	0.0237	0.0000
197	0.0122	0.9626	0.0253	0.0040	0.0116	0.0094
234	0.0000	0.0002	0.9998	0.0000	0.0001	0.0001
262	0.0000	0.0058	0.9942	0.0000	0.0038	0.0038
276	0.0000	0.4388	0.5612	0.0000	0.0350	0.0350
295	0.0000	0.0885	0.9115	0.0000	0.0593	0.0593
301	0.0000	1.0000	0.0000	0.0000	0.0000	0.0000
339	0.0019	0.0079	0.9902	0.0019	0.0031	0.0035
355	0.0000	0.9867	0.0133	0.0000	0.0133	0.0133
363	0.0018	0.0401	0.9581	0.0008	0.0106	0.0104
386	0.0000	1.0000	0.0000	0.0000	0.0000	0.0000
392	0.0000	0.0000	1.0000	0.0000	0.0000	0.0000
398	0.0892	0.9108	0.0000	0.0472	0.0472	0.0000
406	0.0000	1.0000	0.0000	0.0000	0.0000	0.0000
411	0.0000	1.0000	0.0000	0.0000	0.0000	0.0000
448	0.0000	0.2956	0.7044	0.0000	0.0001	0.0001
462	0.0000	0.9994	0.0006	0.0000	0.0004	0.0004
611	0.0000	1.0000	0.0000	0.0000	0.0000	0.0000
618	0.0000	1.0000	0.0000	0.0000	0.0000	0.0000
625	0.0000	1.0000	0.0000	0.0000	0.0000	0.0000
637	0.0000	0.9904	0.0096	0.0000	0.0095	0.0095

Supplementary Table 8.
Site-specific glycosylation of BG505 viral Env, related to **Fig. 2b**.

Glycosite	N+0	N+3	N+203	Standard Error (N+0)	Standard Error (N+3)	Standard Error (N+203)
88	0.0000	0.9621	0.0379	0.0000	0.0227	0.0227
133	0.0000	1.0000	0.0000	0.0000	0.0000	0.0000
137	0.0000	1.0000	0.0000	0.0000	0.0000	0.0000
156	0.0000	0.0000	1.0000	0.0000	0.0000	0.0000
160	0.0000	0.0402	0.9598	0.0000	0.0239	0.0239
185e	0.1677	0.8323	0.0000	0.0409	0.0409	0.0000
185h	0.1656	0.8344	0.0000	0.0568	0.0568	0.0000
197	0.0487	0.9439	0.0074	0.0415	0.0415	0.0035
234	0.0000	0.0000	1.0000	0.0000	0.0000	0.0000
262	0.0000	0.0140	0.9860	0.0000	0.0085	0.0085
276	0.0000	0.5714	0.4286	0.0000	0.0422	0.0422
295	0.0000	0.1632	0.8368	0.0000	0.1051	0.1051
301	0.0000	1.0000	0.0000	0.0000	0.0000	0.0000
339	0.0000	0.0057	0.9943	0.0000	0.0039	0.0039
355	0.0000	0.9938	0.0062	0.0000	0.0044	0.0044
363	0.0019	0.0353	0.9628	0.0010	0.0232	0.0231
386	0.0000	0.9023	0.0977	0.0000	0.0977	0.0977
392	0.0000	0.0509	0.9491	0.0000	0.0509	0.0509
398	0.0524	0.9476	0.0000	0.0524	0.0524	0.0000
406	0.0000	1.0000	0.0000	0.0000	0.0000	0.0000
411	0.0000	0.9355	0.0645	0.0000	0.0645	0.0645
448	0.0000	1.0000	0.0000	0.0000	0.0000	0.0000
462	0.0000	1.0000	0.0000	0.0000	0.0000	0.0000
611	0.0000	1.0000	0.0000	0.0000	0.0000	0.0000
618	ND*	ND	ND	ND	ND	ND
625	ND	ND	ND	ND	ND	ND
637	0.0000	1.0000	0.0000	0.0000	0.0000	0.0000

ND*: not detected

Supplementary Table 9.
Site-specific glycosylation of BG505 SOSIP.664, related to **Fig. 2b**.

Glycosite	N+0	N+3	N+203	Standard Error (N+0)	Standard Error (N+3)	Standard Error (N+203)
88	0.0022	0.5860	0.4119	0.0017	0.0557	0.0557
133	0.0062	0.1100	0.8838	0.0044	0.0328	0.0340
137	0.0257	0.6145	0.3598	0.0109	0.0568	0.0562
156	0.0012	0.0470	0.9517	0.0007	0.0349	0.0349
160	0.0000	0.0702	0.9298	0.0000	0.0459	0.0459
185e	0.1296	0.7878	0.0826	0.0295	0.0407	0.0307
185h	0.0973	0.8649	0.0378	0.0320	0.0376	0.0141
197	0.1164	0.4433	0.4403	0.0564	0.0703	0.0662
234	0.0000	0.0338	0.9662	0.0000	0.0299	0.0299
262	0.0000	0.0066	0.9934	0.0000	0.0028	0.0028
276	0.0000	0.0919	0.9081	0.0000	0.0652	0.0652
295	0.0000	0.0050	0.9950	0.0000	0.0022	0.0022
301	0.0000	0.0000	1.0000	0.0000	0.0000	0.0000
332	0.0833	0.0469	0.8698	0.0833	0.0402	0.0886
339	0.0166	0.0605	0.9229	0.0091	0.0587	0.0585
355	0.0037	0.6625	0.3338	0.0024	0.0706	0.0706
363	0.0010	0.0144	0.9846	0.0005	0.0065	0.0065
386	0.0000	0.1194	0.8806	0.0000	0.0407	0.0407
392	0.0000	0.0865	0.9135	0.0000	0.0213	0.0213
398	0.0242	0.6819	0.2939	0.0056	0.0676	0.0635
406	0.0000	0.8502	0.1498	0.0000	0.0263	0.0263
411	0.0001	0.1053	0.8946	0.0000	0.0410	0.0410
448	0.0000	0.0000	1.0000	0.0000	0.0000	0.0000
462	0.0000	0.9151	0.0849	0.0000	0.0398	0.0398
611	0.0000	1.0000	0.0000	0.0000	0.0000	0.0000
618	0.2435	0.7449	0.0117	0.1009	0.1003	0.0099
625	0.5410	0.1681	0.2910	0.1078	0.0557	0.0956
637	0.0237	0.6576	0.3187	0.0081	0.0808	0.0834

Supplementary Table 10.
Site-specific glycosylation of B41 ΔCT, related to **Fig. 2c**.

Glycosite	N+0	N+3	N+203	Standard Error (N+0)	Standard Error (N+3)	Standard Error (N+203)
88	0.0000	0.9978	0.0022	0.0000	0.0016	0.0016
137	0.0344	0.9656	0.0000	0.0150	0.0150	0.0000
140	0.1165	0.8835	0.0000	0.0350	0.0350	0.0000
143a	0.0048	0.9952	0.0000	0.0021	0.0021	0.0000
156	0.0026	0.0295	0.9680	0.0025	0.0080	0.0080
160	0.0000	0.0161	0.9839	0.0000	0.0093	0.0093
186e	0.0001	0.9961	0.0038	0.0001	0.0015	0.0014
187	0.1664	0.8303	0.0033	0.0207	0.0206	0.0019
197	0.0009	0.9961	0.0030	0.0006	0.0021	0.0021
234	0.0000	0.0011	0.9989	0.0000	0.0011	0.0011
241	0.0003	0.0482	0.9515	0.0003	0.0128	0.0128
262	0.0000	0.0008	0.9992	0.0000	0.0005	0.0005
276	0.0000	0.0153	0.9847	0.0000	0.0063	0.0063
295	0.0000	0.0000	1.0000	0.0000	0.0000	0.0000
301	0.0000	0.9404	0.0596	0.0000	0.0304	0.0304
332	0.0117	0.0242	0.9640	0.0103	0.0125	0.0188
339	0.0012	0.9612	0.0377	0.0004	0.0165	0.0162
355	0.0000	0.9902	0.0098	0.0000	0.0055	0.0055
362	0.0166	0.0245	0.9590	0.0090	0.0198	0.0219
386	0.0000	0.0099	0.9901	0.0000	0.0055	0.0055
392	0.0000	0.4426	0.5574	0.0000	0.0222	0.0222
396	0.0000	1.0000	0.0000	0.0000	0.0000	0.0000
413	0.0045	0.0037	0.9918	0.0025	0.0015	0.0032
448	0.0000	0.0255	0.9745	0.0000	0.0087	0.0087
463	0.0000	1.0000	0.0000	0.0000	0.0000	0.0000
611	0.0174	0.9826	0.0000	0.0137	0.0137	0.0000
616	0.0000	0.9816	0.0184	0.0000	0.0105	0.0105
625	0.0144	0.9856	0.0000	0.0143	0.0143	0.0000
637	0.0057	0.9728	0.0215	0.0038	0.0131	0.0115

Supplementary Table 11.
Site-specific glycosylation of B41 viral Env, related to **Fig. 2c**.

Glycosite	N+0	N+3	N+203	Standard Error (N+0)	Standard Error (N+3)	Standard Error (N+203)
88	0.0000	1.0000	0.0000	0.0000	0.0000	0.0000
137	ND*	ND	ND	ND	ND	ND
140	ND	ND	ND	ND	ND	ND
143a	0.0000	1.0000	0.0000	0.0000	0.0000	0.0000
156	0.0000	0.0000	1.0000	0.0000	0.0000	0.0000
160	0.0000	0.0479	0.9521	0.0000	0.0473	0.0473
186e	0.0000	1.0000	0.0000	0.0000	0.0000	0.0000
187	0.0326	0.9669	0.0005	0.0103	0.0103	0.0003
197	0.0000	1.0000	0.0000	0.0000	0.0000	0.0000
234	0.0000	0.0000	1.0000	0.0000	0.0000	0.0000
241	0.0000	0.0897	0.9103	0.0000	0.0892	0.0892
262	0.0000	0.0391	0.9609	0.0000	0.0134	0.0134
276	0.0000	0.0430	0.9570	0.0000	0.0189	0.0189
295	0.0000	0.0000	1.0000	0.0000	0.0000	0.0000
301	0.0000	1.0000	0.0000	0.0000	0.0000	0.0000
332	0.0000	0.0420	0.9580	0.0000	0.0420	0.0420
339	0.0000	0.8983	0.1017	0.0000	0.0498	0.0498
355	0.0000	0.9999	0.0001	0.0000	0.0001	0.0001
362	0.0030	0.0364	0.9606	0.0018	0.0129	0.0131
386	0.0000	0.0000	1.0000	0.0000	0.0000	0.0000
392	0.0000	0.4730	0.5270	0.0000	0.1166	0.1166
396	0.0000	1.0000	0.0000	0.0000	0.0000	0.0000
413	0.0000	0.0031	0.9969	0.0000	0.0030	0.0030
448	0.0000	0.0000	1.0000	0.0000	0.0000	0.0000
463	0.0000	1.0000	0.0000	0.0000	0.0000	0.0000
611	0.0000	0.9993	0.0007	0.0000	0.0007	0.0007
616	0.0000	1.0000	0.0000	0.0000	0.0000	0.0000
625	0.0000	1.0000	0.0000	0.0000	0.0000	0.0000
637	0.0006	0.9688	0.0306	0.0005	0.0191	0.0189

ND*: not detected

Supplementary Table 12.
Site-specific glycosylation of B41 SOSIP.664, related to **Fig. 2c**.

Glycosite	N+0	N+3	N+203	Standard Error (N+0)	Standard Error (N+3)	Standard Error (N+203)
88	0.0000	0.7669	0.2331	0.0000	0.0282	0.0282
137	0.0000	0.2222	0.7778	0.0000	0.1079	0.1079
140	0.0140	0.8677	0.1183	0.0138	0.1089	0.0953
143a	0.0000	0.8090	0.1910	0.0000	0.1038	0.1038
156	0.0000	0.0306	0.9694	0.0000	0.0172	0.0172
160	0.0000	0.0465	0.9535	0.0000	0.0395	0.0395
186e	0.0000	0.9006	0.0994	0.0000	0.0305	0.0305
187	0.1989	0.2082	0.5929	0.0376	0.0551	0.0658
197	0.0000	0.8078	0.1922	0.0000	0.0494	0.0494
234	0.0004	0.0799	0.9196	0.0002	0.0271	0.0273
241	0.0000	0.1735	0.8264	0.0000	0.0251	0.0251
262	0.0002	0.0389	0.9609	0.0002	0.0265	0.0265
276	0.0000	0.0252	0.9748	0.0000	0.0135	0.0135
295	0.0000	0.0000	1.0000	0.0000	0.0000	0.0000
301	0.0000	0.0000	1.0000	0.0000	0.0000	0.0000
332	0.0000	0.0179	0.9821	0.0000	0.0179	0.0179
339	0.0000	0.8946	0.1054	0.0000	0.0385	0.0385
355	0.0000	0.8690	0.1310	0.0000	0.0330	0.0330
362	0.0048	0.0763	0.9189	0.0023	0.0191	0.0192
386	0.0955	0.0127	0.8919	0.0310	0.0029	0.0292
392	0.0000	0.5570	0.4430	0.0000	0.0391	0.0391
396	0.0000	1.0000	0.0000	0.0000	0.0000	0.0000
413	0.0008	0.0111	0.9881	0.0005	0.0056	0.0058
448	0.0000	0.0440	0.9560	0.0000	0.0272	0.0272
463	0.0000	0.7082	0.2918	0.0000	0.1408	0.1408
611	0.3680	0.4157	0.2163	0.1086	0.0988	0.0765
616	0.0497	0.7772	0.1731	0.0236	0.0704	0.0652
625	0.4611	0.2767	0.2622	0.0887	0.0654	0.0686
637	0.0434	0.2710	0.6856	0.0238	0.0553	0.0515
